# Determining the Joint Effect of Obesity and Diabetes on All-Cause Mortality and Cardiovascular-Related Mortality following an Ischemic Stroke

**DOI:** 10.1155/2018/4812712

**Published:** 2018-08-09

**Authors:** Colleen Bauza, Renee' Martin, Sharon D. Yeatts, Keith Borg, Gayenell Magwood, Anbesaw Selassie, Marvella E. Ford

**Affiliations:** ^1^Department of Public Health Sciences, Medical University of South Carolina, Charleston, SC, USA; ^2^Department of Health Informatics, Johns Hopkins All Children's Hospital, St. Petersburg, FL, USA; ^3^Department of Emergency Medicine, Medical University of South Carolina, Charleston, SC, USA; ^4^Department of Nursing, Medical University of South Carolina, Charleston, SC, USA

## Abstract

Although obesity and diabetes mellitus, or diabetes, are independently associated with mortality-related events (*e.g.,* all-cause mortality and cardiovascular-related mortality) following an ischemic stroke, little is known about the joint effect of obesity and diabetes on mortality-related events following an ischemic stroke. The aim of this study is to evaluate the joint effect of obesity and diabetes on mortality-related events in subjects with a recent ischemic stroke. Data from the multicenter Prevention Regimen for Effectively Avoiding Second Strokes (PRoFESS) trial was analyzed for this study. The joint effect of obesity and diabetes on mortality-related events was estimated via Cox proportional hazards regression models. No difference in the hazard of all-cause mortality following an ischemic stroke was observed between obese subjects with diabetes and underweight/normal-weight subjects without diabetes. In contrast, obese subjects with diabetes had an increased hazard of cardiovascular-related mortality following an ischemic stroke compared with underweight/normal-weight subjects without diabetes. Additionally, there was evidence of an attributable proportion due to interaction as well as evidence of a highly statistically significant interaction on the multiplicative scale for cardiovascular-related mortality. In this clinical trial cohort of ischemic stroke survivors, obesity and diabetes synergistically interacted to increase the hazard of cardiovascular-related mortality.

## 1. Introduction

Although obesity and diabetes are established independent risk factors for ischemic stroke [1], their joint effect on stroke risk is not well explored. In the only published study of the joint effect of obesity and diabetes on stroke risk, Olofindayo* et al.* [2] determined that the effect of central obesity, measured by waist circumference, on the risk of a stroke differed by diabetes status (*e.g.,* having or not having a prior diagnosis of diabetes). Specifically, they found that individuals with both central obesity and diabetes had an increased risk for stroke greater than the sum (and product) of the risk of individuals with either central obesity or diabetes [2].

To date, there is no known study on the joint effect, or interactive effects, of these factors on mortality-related events (*e.g.,* all-cause mortality and cardiovascular-related mortality) following an ischemic stroke. It is known, however, that obesity and diabetes are independently associated with mortality-related events following an ischemic stroke. The reported effects of obesity on mortality-related events following an ischemic stroke have been conflicting. Whereas studies of the general population have found that increasing weight increases the risk of mortality [3–5], a number of observational studies have reported that obesity is associated with a decreased risk of both all-cause [6–11] and cardiovascular-related [7, 12, 13] mortality following a stroke; this apparent discrepancy is referred to as the obesity paradox [6–13]. Several investigators have questioned the validity of the obesity paradox, citing methodological issues (*e.g.,* the measurement of obesity, treatment, and/or selection bias due to the study population) or residual confounding as potential explanations [14–16]. In contrast to the conflicting reported effects on obesity and mortality-related events following a stroke, prior studies have established that diabetes is associated with higher risk of all-cause mortality [17–20] and cardiovascular-related mortality [19] following a stroke.

The current study assumed that the conflicting findings in the obesity and diabetes literature may be due to a joint effect of obesity and diabetes which has not been examined previously. The pathogenesis of stroke is a heterogeneous process that involves molecular, cellular, neuronal, individual, and environmental factors [21]. Because diabetes is related to obesity [22, 23], diabetes may potentially modify the inflammatory effects of body mass on risk of mortality-related events after an ischemic stroke. If so, elucidation of the joint effect of obesity and diabetes may help identify high-risk subgroups and provide new insights into underlying mechanisms.

Although previous studies have shown evidence that obesity and diabetes are independently associated with mortality-related events in a stroke population, there is a lack of research on the joint effect of obesity and diabetes on mortality-related events following an ischemic stroke. The current research has tremendous public health relevance given the increasing prevalence of obesity and the expected increase in prevalence of diabetes in the US and internationally. The objective of this study was to determine the joint effect of obesity and diabetes on the risk of mortality-related events following an ischemic stroke in a cohort of ischemic stroke survivors.

## 2. Materials and Methods

### 2.1. Study Population

The population of interest for this work is ischemic stroke survivors. Previous work looking at all-cause mortality at 1 year in ischemic stroke patients [24] showed that there was insufficient evidence to determine that the effect of obesity differed by diabetes status. This study was limited because the study population included acute ischemic stroke patients with at least a moderate stroke severity who met study eligibility. The data source for the current study is the PRoFESS trial, a double-blind 2-by-2 factorial trial (ClinicalTrials.gov number: NCT00153062) [25]. The objective of the PRoFESS trial was to determine if extended-release dipyridamole and aspirin were superior to clopidogrel and if telmisartan was superior to placebo in preventing a recurrent stroke in subjects who were recently diagnosed with an ischemic stroke [25]. Details of the scientific rationale, eligibility requirements, and baseline characteristics of the PRoFESS subjects have been published elsewhere [25–28]. Briefly, 20,332 subjects were enrolled in PRoFESS between September 2003 and July 2006 at 695 study centers in 35 countries and were followed up for a median time of 2.4 years (range: 1.5-4.4 years) from randomization [26–28]. To be eligible, subjects had to be at least 55 years old and were randomized within 90 days of experiencing an ischemic stroke or had to be between 50 and 54 years old, have at least two additional risk factors, and were randomized between 90 and 120 days after experiencing an ischemic stroke [25].

### 2.2. Exposures

BMI and diabetes are the exposures of interest for the current study and were defined based on information identified at the baseline visit. Typically, BMI is defined as weight in kilograms per the square of height in meters (kg/m^2^) [29]. Subjects were categorized into underweight/normal weight (BMI < 25.0 kg/m^2^), overweight (BMI 25.0-29.9 kg/m^2^), and obese (BMI ≥ 30.0 kg/m^2^) based on the published guidelines of the World Health Organization [29]. Prior diagnosis of diabetes (yes, no) was measured as a summary variable. Diagnosis of diabetes was ascertained using source documentation (*i.e., *the subject's medical record).

### 2.3. Outcomes

All-cause mortality, defined as death due to any cause, and cardiovascular-related mortality, defined as death due to stroke (*i.e.,* ischemic, hemorrhagic, or uncertain cause), myocardial infarction, hemorrhage excluding intracranial bleeding, and other vascular causes [28], were the primary outcomes for this research. At the completion of the PRoFESS trial, 1,495 subjects had died from all causes, and 894 subjects had died due to cardiovascular-related causes. These dependent variables were confirmed by the PRoFESS trial's Adjudication and Assessment Committee [25].

### 2.4. Statistical Analysis

Subjects with missing obesity and diabetes information were excluded from the analysis. All subjects were followed up from the date of enrollment until the date of death, until they were lost to follow-up, or until the end of the clinical trial, whichever occurred first. Baseline characteristics were presented as a median and interquartile range for continuous variables and as a number and proportion for categorical variables.

Cox proportional hazards (CPH) regression models were utilized to model the relationship between all-cause and cardiovascular-related mortality and exposures of BMI and diabetes as defined above; adjusted hazard ratios (HR) and 95% confidence intervals (CI) were constructed according to a common reference category of underweight/normal-weight subjects without diabetes. A multivariable CPH regression model for each mortality-related event was fit including prespecified variables that were forced into the final model in addition to potential confounders, which are shown in**Table 1**. Due to their known prognostic value, age, gender, race/ethnicity, baseline stroke severity, ischemic stroke subtype, and treatment assignment were forced into the final models for both mortality-related events. A variable was considered as a potential confounder if it was significant univariately at the *α* = 0.25 level or its presence resulted in at least a 10% difference in the effect measure between the crude and adjusted estimates. Backward selection using the likelihood ratio test was used to obtain the final models that included the significant confounders. The proportional hazards assumption was assessed using Schoenfeld residuals and time-dependent covariates [30]. For continuous variables, linearity in the log hazard was evaluated by assessing the cumulative martingale residuals. Additionally, multicollinearity between covariates was assessed by calculating individual variance inflation factors for each of the exposure variables and the potential confounders.

The joint effect of BMI and diabetes was examined on both the multiplicative and additive scales in relation to the hazard of all-cause mortality (and cardiovascular-related mortality) following an ischemic stroke. The likelihood ratio test was used to determine the significance of the cross-product interaction term between BMI and diabetes. Additionally, the joint effect on the additive scale, or the biologic interaction, was evaluated by two indices: the relative excess risk because of the interaction (RERI) and the attributable proportion because of the interaction (AP).[31] RERI is an estimate of the excess risk attributable to the joint effect of obesity and diabetes and AP is defined as the proportion of risk attributable to the joint effect of obesity and diabetes [31]. These indices, along with their 95% CIs, were constructed using the approach of Li and Chambless [32]. A value of 0 indicates that there is no biologic interaction present [32, 33].

All statistical tests were two-sided and used an alpha level of 0.05 with the exception of the joint effect on the multiplicative scale. For the joint effect on the multiplicative scale, statistical significance was defined at an alpha level of 0.10 rather than 0.05, because clinical trials are not designed to detect a joint effect but only a main effect [34]. Statistical analyses were conducted using SAS software package version 9.4 (SAS Institute, Cary, NC). Institutional Review Board's approval for this analysis was obtained from the Medical University of South Carolina.

## 3. Results

### 3.1. Baseline Characteristics of the PRoFESS Study Sample

Of the 20,332 subjects enrolled in the PRoFESS trial, BMI or diabetes information was not available for 86 subjects (0.42%). As a result, data from 20,246 subjects were analyzed in the current study. Baseline characteristics and the number of deaths due to all causes and due to cardiovascular-related causes observed according to BMI category and diabetes status are shown in**Table 2**. Among these 20,246 subjects with complete BMI and diabetes information, few subjects were obese (20.96%) and had a prior diagnosis of diabetes (28.22%). The majority of subjects were male (64.09%), older than 65 years (54.86%), and white (57.30%), had a history of hypertension (74.03%), had no prior stroke or TIA (75.42%), were former/never smokers (78.81%), and had mild neurological severity for their qualifying stroke defined as a NIHSS < 8 (93.35%). Among subjects without diabetes, compared with underweight/normal-weight subjects, obese subjects were more likely to have the following characteristics: female, white, history of hypertension, history of coronary artery disease, history of congestive heart failure, history of myocardial infarction, history of hyperlipidemia, former/never smokers, and sedentary. Similarly, among subjects with diabetes, compared with underweight/normal-weight subjects, obese subjects were also more likely to be female and white, have a history of hypertension, have a history of coronary artery disease, have a history of congestive heart failure, have a history of myocardial infarction, have a history of hyperlipidemia, be former/never smokers, and be sedentary. In contrast, among subjects with diabetes, obese subjects were more likely to have a mild neurological severity for their qualifying stroke.

### 3.2. All-Cause Mortality following an Ischemic Stroke

The adjusted joint effects of BMI categories and diabetes status on all-cause mortality are shown in**Table 3.** Obese subjects with diabetes had a similar hazard of all-cause mortality following an ischemic stroke compared with the reference group or underweight/normal-weight subjects without diabetes (HR: 0.98, 95% CI: 0.80, 1.21). There was insufficient evidence to declare an interaction between obesity and diabetes on either the multiplicative (*P*_interaction_=0.1440) or the additive scale (RERI: -0.019, 95% CI: -0.319, 0.280; AP: -0.020, 95% CI: -0.112, 0.073).

### 3.3. Cardiovascular-Related Mortality following an Ischemic Stroke

The adjusted joint effects of BMI categories and diabetes status on cardiovascular-related mortality are shown in**Table 4**. Obese subjects with diabetes had an increased hazard of cardiovascular-related mortality following an ischemic stroke compared with underweight/normal-weight subjects without diabetes (HR: 1.44, 95% CI: 1.12, 1.86). On the multiplicative scale, there was a significant joint effect of obesity and diabetes (*P*_interaction_= 0.0049). In addition, a significant additive interactive effect between obesity and diabetes on cardiovascular-related mortality was indicated by the significant AP estimate and its confidence interval (AP: 0.258, 95% CI: 0.155, 0.361). In contrast, there was insufficient evidence of an additive interaction for the RERI (0.372, 95% CI: -0.064, 0.808).

## 4. Discussion

Although obesity [6–13] and diabetes [17–20] are independently associated with mortality-related events following an ischemic stroke, this was the first study to explore the potential joint effect of obesity and diabetes on mortality-related events following an ischemic stroke on the additive and multiplicative scales. Studying joint effects can identify susceptible subgroups of individuals that would potentially benefit from effective interventions [35]. Joint effects on the additive scale are important public health indices [35, 36] because they have been suggestive of a biological interaction or an underlying causal mechanism [35, 36].

In the current study, there was insufficient evidence to determine a joint effect of obesity and diabetes on all-cause mortality on either the additive or multiplicative scales. However, compared with underweight/normal-weight subjects without diabetes, obese subjects with diabetes were approximately 44% (HR: 1.44, 95% CI: 1.12, 1.86) more likely to die from cardiovascular-related causes following an ischemic stroke. Although the combined effect of obesity and diabetes did not exceed the sum of the separate effects of obesity and diabetes (RERI: 0.372, 95% CI: -0.064, 0.808), approximately 26.0% of the cardiovascular-related deaths following an ischemic stroke in this cohort of ischemic stroke survivors could be attributed to the joint effect of obesity and diabetes (AP: 0.258, 95% CI: 0.155, 0.361).

The World Health Organization (WHO) expert consultation recently advocated for different BMI cut-off limits for individuals of Asian race/ethnicity [37]. As a result, subgroup analyses were performed to investigate the consistency in results among Asians using the WHO standard BMI and the Asia Pacific Guidelines from the WHO. Conclusions were not substantively altered in the subgroup of Asian race/ethnicity when the Asia Pacific Guidelines for BMI categories were applied.

To account for the potential residual confounding effect by smoking, subgroup analyses were conducted to determine the consistency in results among subjects who were current smokers and subjects who were former/never smokers (**Supplementary Tables **1-4). Although the conclusions did not substantively differ in the subgroups of former/never smokers or current smokers, there are several noteworthy findings. For all-cause mortality, the point estimate for obese subjects with diabetes compared to underweight/normal-weight subjects without diabetes was increased among current smokers. Furthermore, among current smokers, approximately 16.8% of the deaths from any cause following an ischemic stroke in this cohort of ischemic stroke survivors could be attributed to the joint effect of obesity and diabetes (AP: 0.168, 95% CI: 0.006, 0.330).

The exact mechanisms by which obesity and diabetes increase the hazard of cardiovascular-related mortality following an ischemic stroke cannot be determined from the present study. Given that obesity and diabetes are major risk factors for cardiovascular morbidity and mortality and are additionally associated with hypertension, dyslipidemia, and elevated levels of fibrinogen and C-reactive protein, other risk factors for cardiovascular morbidity and mortality [22, 23, 38], future studies can focus on understanding the mechanisms for this finding. Hence, it is hypothesized that these mechanisms are multifactorial and involve molecular, cellular, neuronal, individual, and environmental factors [21].

### 4.1. Limitations and Strengths

The present study included several limitations, one of which was survivorship bias related to study sample selection. This study's sample consisted of a cohort of individuals who survived a period of time (median time = 15 days) following onset of a qualifying stroke and who met specific inclusion criteria of the PRoFESS trial. Hence, there was a potential for selection bias because individuals with a more severe neurologic deficit at the time of the qualifying stroke may have not been included in the PRoFESS trial.

Other limitations identified were associated with the exposure variables of obesity and diabetes. The data available for the study measured weight only at randomization, and thus the potential impact of weight change over the course of the follow-up period could not be assessed. Although weight loss after a stroke is relatively common, Jönsson* et al.* [39] noted that the median weight loss four months following a stroke was only 0.6 kg (or 1.32 lbs) in a cohort of first-time stroke patients. Hence, in the present study, it was assumed that the median amount of weight that subjects may have lost between ischemic stroke onset and baseline BMI assessment would result in subjects maintaining a relatively consistent BMI or BMI category between these two time periods. In addition, the PRoFESS trial data did not differentiate between type 1 and type 2 diabetes. However, as type 2 diabetes accounts for approximately 95% of all diagnosed cases of diabetes in the US [40, 41], it may be assumed that the majority of diabetes cases in the PRoFESS trial were type 2 diabetes.

Due to the restrictive inclusion criteria of the PRoFESS trial, the results of the present study may not be generalizable to all ischemic stroke survivors. For example, individuals were excluded if they had a severe disability after the qualifying stroke [25]. Furthermore, the PRoFESS trial was not designed to answer the research questions of the present study. Examining joint effects, or interactions, is challenging because tests for interactions are typically underpowered [42]. Hence, it is critical to utilize a national or international ischemic stroke registry that would provide sufficient resources and power for future studies to address these research questions. Additionally, the potential for reverse causation cannot be excluded. Future research could be performed to determine if the results demonstrated among a cohort of ischemic stroke survivors with a median follow-up time of 2.4 years would be similar to the results among a cohort of acute ischemic stroke subjects with a longer follow-up time.

Despite some limitations, the present study includes several notable strengths. First, this study utilized data from a large clinical trial with prospective ascertainment of the dependent variables of interest. Further, the rigorous data collection of the PRoFESS trial reduced information bias. Rather than relying on subjects self-reporting their medical history, the PRoFESS trial utilized source documents to verify subjects' medical history. Additionally, the use of clinical trial data ensured that strict study procedures were followed, which minimized the potential bias from incorrect documentation of the trial's outcomes.

This study was the first to examine the joint effects, or the interactive effects, of BMI and diabetes on mortality-related events following an ischemic stroke on the additive and multiplicative scales. Additionally, the results of this study provide evidence for generating hypotheses for future studies investigating how BMI and diabetes could potentially interact with one another to affect the risk of mortality-related events following an ischemic stroke. These results could also be used by future investigators to develop interventions focused on reducing the burden of all-cause and cardiovascular-related mortality following onset of an ischemic stroke.

### 4.2. Clinical Relevance

The American Heart Association and the American Stroke Association recommend using BMI to diagnose obesity [43]. However, BMI may not be the best diagnostic tool to measure obesity because this diagnostic tool is unable to differentiate between body fat and lean mass [44]. It has recently been hypothesized that there are different phenotypes of obesity, namely, metabolically healthy obese and metabolically unhealthy obese [45]. Thus, it is critical to determine new diagnostic tools capable of differentiating risk of poor outcomes following an ischemic stroke based on BMI (or waist circumference or waist-to-hip ratio) [14, 44] and metabolic health.

### 4.3. Public Health Relevance

Obesity and diabetes are highly prevalent in both the general US and international populations [41, 46–48] as well as among individuals who have been diagnosed with a stroke [43]. Although the prevalence of obesity and diabetes varies by region and country, it is estimated that between 18% and 44% of individuals who previously had an ischemic stroke are obese, and between 25% and 45% of individuals who previously had an ischemic stroke have diabetes [43]. Despite the high prevalence of these risk factors among stroke survivors, the current guidelines from the American Heart Association and American Stroke Association only recommend that all individuals who are diagnosed with an ischemic stroke be screened for diabetes and obesity [43]. The guidelines no longer recommend weight reduction for individuals with a BMI over 25 kg/m^2^ due to the unexpected relationship between obesity and prognosis after stroke and the null results of a weight loss intervention [43]. Thus, it is important to focus research on understanding the mechanism by which diabetes modifies the relationship between BMI and mortality-related events following an ischemic stroke in order to develop more focused guidelines and interventions to reduce mortality rates for individuals with these risk factors.

### 4.4. Future Directions

The current study included a post hoc analysis of data from the PRoFESS trial. Results from this study add valuable information to the literature regarding post-ischemic-stroke outcomes. Specifically, the findings suggest that obese individuals with diabetes have an increased hazard of cardiovascular-related mortality following an ischemic stroke compared with underweight/normal-weight individuals without diabetes. Thus, results from the current study suggest that future interventions should focus resources on obese individuals with diabetes in order to reduce the excess burden of cardiovascular-related mortality in this group. Additionally, future population-based cohort studies are needed to examine whether the effect of obesity, measured by BMI or another diagnostic tool, on mortality-related events following an ischemic stroke differs by diabetes status.

## Figures and Tables

**Table 1:** Variables and definitions of prespecified variables and potential confounders for analysis.

**Variables**	**Definition**
**Pre-Specified Variables**	

Age *∗*†	< 65 years, ≥ 65 years

Gender *∗*†	Male, Female

Race/ethnicity *∗*†	non-Hispanic White, non-Hispanic Black, Hispanic, Asian, Other

Treatment assignment *∗*†	Aspirin + extended release dipyridamole/telmisartan, Clopidogrel/telmisartan, Aspirin + extended release dipyridamole/placebo, Clopidogrel/placebo

Qualifying stroke neurological severity *∗*†	Mild (NIHSS < 8), Moderate/Severe (NIHSS ≥ 8)

Ischemic stroke sub-type *∗*†	Large-artery atherosclerosis, Cardioembolism, Small-artery occlusion, Other, Undetermined

**Potential Confounders**	

Baseline systolic blood pressure *∗*†	in mmHg

History of previous stroke or TIA *∗*†	Yes, No

History of previous myocardial infarction *∗*†	Yes, No

History of atrial fibrillation *∗*	Yes, No

History of congestive heart failure *∗*	Yes, No

History of coronary artery disease *∗*†	Yes, No

History of hypertension *∗*†	Yes, No

History of hyperlipidemia †	Yes, No

Smoking status *∗*†	Current smoker, Former/Never smoker

Regular alcohol consumption *∗*	At least 1 drink/week, No alcohol consumption

Average physical activity prior to qualifying stroke *∗*†	Sedentary: walking <1 mile/day, Some physical activity: 20-30 minutes, 3 times/week, Intense physical activity: > 30 minutes, > 3 times/week

*∗* indicates potential confounder for all-cause mortality; † indicates potential confounder for cardiovascular-related mortality.

**Table 2:** Baseline characteristics of 20,246 ischemic stroke survivors based on BMI categories and diabetes, the PRoFESS trial.

**Characteristic**		**No diabetes**		
	All participants	Underweight/ normal weight	Overweight	Obese
	No. (%)	No. (%)	No. (%)	No. (%)

**No. of participants ** ^**a**^	20246	5958	5881	2693
No. of participants who died from all causes	1485	495	337	126
No. of participants who died from cardiovascular-related causes	887	288	190	67
Diabetes				
No	14532 (71.78)			
Yes	5714 (28.22)			
BMI categories				
Underweight/normal weight	7864 (38.84)			
Overweight	8138 (40.20)			
Obese	4244 (20.96)			
BMI (kg/m^2^) (median, IQR)	26.1 (23.6-29.3)	22.9 (21.3-24.1)	27.1 (26.0-28.3)	32.5 (31.1-35.2)
**Sociodemographic Characteristics**				
Age				
≥ 65 years	11107 (54.86)	3558 (59.72)	3288 (55.91)	1364 (50.65)
Gender				
Male	12976 (64.09)	3843 (64.50)	4025 (68.44)	1450 (53.84)
Race/ethnicity				
White	11600 (57.3)	2834 (47.57)	3972 (67.54)	2118 (78.65)
Black	804 (3.97)	146 (2.45)	177 (3.01)	149 (5.53)
Asian	6645 (32.82)	2712 (45.52)	1416 (24.08)	236 (8.76)
Hispanic	987 (4.88)	223 (3.74)	271 (4.61)	148 (5.50)
Other	210 (1.04)	43 (0.72)	45 (0.77)	42 (1.56)
**Clinical Characteristics**				
Qualifying stroke neurological severity				
Mild	18896 (93.35)	5512 (92.53)	5571 (94.76)	2555 (94.88)
Moderate/Severe	1347 (6.65)	445 (7.47)	308 (5.24)	138 (5.11)
Qualifying stroke subtype				
Large vessel atherosclerosis	5789 (28.61)	1851 (31.09)	1594 (27.12)	639 (23.75)
Cardioembolic	369 (1.82)	111 (1.86)	126 (2.14)	64 (2.38)
Small vessel disease	10530 (52.04)	2999 (50.38)	2986 (50.80)	1399 (52.01)
Other	411 (2.03)	109 (1.83)	133 (2.26)	66 (2.45)
Unknown	3135 (15.49)	883 (14.83)	1039 (17.68)	522 (19.41)
Baseline systolic blood pressure (median, IQR)	142 (130-156)	141 (130-155)	142 (131-156)	143 (131-157)
Treatment group				
A †	5024 (25.01)	1499 (25.36)	1464 (25.07)	672 (25.17)
B ‡	5004 (24.91)	1442 (24.39)	1472 (25.21)	680 (25.47)
C §	5041 (25.09)	1462 (24.73)	1461 (25.02)	636 (23.82)
D | |	5019 (24.99)	1509 (25.52)	1442 (24.70)	682 (25.54)
**Risk Factors and Comorbidities**				
History of previous stroke or TIA				
Yes	4975 (24.58)	1482 (24.89)	1337 (22.74)	602 (22.37)
History of previous myocardial infarction				
Yes	1362 (6.73)	292 (4.90)	382 (6.50)	199 (7.39)
History of atrial fibrillation				
Yes	538 (2.66)	165 (2.77)	183 (3.11)	89 (3.30)
History of congestive heart failure				
Yes	532 (2.63)	113 (1.90)	143 (2.43)	121 (4.49)
History of coronary artery disease				
Yes	3296 (16.28)	729 (12.24)	994 (16.90)	534 (19.83)
History of hypertension				
Yes	14987 (74.03)	3788 (65.58)	4264 (72.52)	2152 (79.91)
History of hyperlipidemia				
Yes	9453 (46.76)	2245 (37.73)	2730 (46.48)	1366 (50.89)
Smoking status				
Current smoker	4288 (21.19)	1682 (28.24)	1217 (20.70)	406 (15.09)
Regular alcohol consumption				
≥ 1 drink/week	7206 (35.45)	2217 (37.22)	2434 (41.39)	959 (35.64)
Average physical activity prior to qualifying stroke				
Sedentary	7209 (35.83)	1824 (30.76)	1865 (31.95)	1175 (44.01)
Some physical activity	6446 (32.04)	1979 (33.37)	1937 (33.18)	786 (29.44)
Intense physical activity	6465 (32.13)	2127 (35.87)	2036 (34.87)	709 (26.55)

**Characteristic**		**Diabetes**		
		Underweight/ normal weight	Overweight	Obese
		No. (%)	No. (%)	No. (%)

**No. of participants** ^**∗**^		1906	2257	1551
No. of participants who died from all causes		219	180	128
No. of participants who died from cardiovascular-related causes		136	111	95
Diabetes				
No				
Yes				
BMI categories				
Underweight/normal-weight				
Overweight				
Obese				
BMI (kg/m^2^) (median, IQR)		23.0 (21.5-24.0)	27.2 (26.0-28.4)	33.1 (31.3-36.3)
**Sociodemographic Characteristics**				
Age				
≥ 65 years		1000 (52.47)	1178 (52.19)	719 (46.36)
Gender				
Male		1271 (66.68)	1530 (67.79)	857 (55.25)
Race/ethnicity				
White		482 (25.29)	1106 (49.00)	1088 (70.15)
Black		51 (2.68)	118 (5.23)	163 (10.51)
Asian		1277 (67.00)	847 (37.53)	157 (10.12)
Hispanic		82 (4.30)	159 (7.04)	104 (6.71)
Other		14 (0.73)	27 (1.20)	39 (2.51)
**Clinical Characteristics**				
Qualifying stroke neurological severity				
Mild		1718 (90.14)	2083 (92.29)	1457 (93.94)
Moderate/Severe		188 (9.86)	174 (7.71)	94 (6.06)
Qualifying stroke subtype				
Large vessel atherosclerosis		663 (34.78)	654 (28.99)	388 (25.02)
Cardioembolic		21 (1.10)	27 (1.20)	20 (1.29)
Small vessel disease		1036 (54.35)	1237 (54.83)	873 (56.29)
Other		17 (0.89)	41 (1.82)	45 (2.90)
Unknown		169 (8.87)	297 (13.16)	225 (14.51)
Baseline systolic blood pressure (median, IQR)		142 (130-157)	144 (132-158)	144 (131-158)
Treatment group				
A †		434 (22.90)	588 (26.24)	367 (23.97)
B ‡		509 (26.86)	524 (23.38)	377 (24.62)
C §		497 (26.23)	583 (26.02)	402 (26.26)
D | |		455 (24.01)	546 (24.36)	385 (25.15)
**Risk Factors and Comorbidities**				
History of previous stroke or TIA				
Yes		547 (28.70)	621 (27.55)	386 (24.89)
History of previous myocardial infarction				
Yes		105 (5.51)	206 (9.14)	178 (11.48)
History of atrial fibrillation				
Yes		27 (1.42)	34 (1.51)	40 (2.58)
History of congestive heart failure				
Yes		34 (1.78)	52 (2.30)	69 (4.45)
History of coronary artery disease				
Yes		252 (13.22)	433 (19.18)	354 (22.82)
History of hypertension				
Yes		1481 (77.70)	1901 (84.23)	1401 (90.33)
History of hyperlipidemia				
Yes		882 (46.35)	1271 (56.39)	959 (61.91)
Smoking status				
Current smoker		362 (18.99)	427 (18.93)	194 (12.52)
Regular alcohol consumption				
≥ 1 drink/week		467 (24.50)	696 (30.85)	404 (26.06)
Average physical activity prior to qualifying stroke				
Sedentary		694 (36.70)	896 (39.86)	755 (48.96)
Some physical activity		592 (31.31)	696 (30.95)	456 (29.57)
Intense physical activity		605 (31.99)	656 (29.18)	331 (21.47)

*∗* indicates that 86 participants were excluded due to missing BMI or diabetes information; † indicates Aspirin + Extended Release Dipyridamole/Telmisartan; ‡ indicates Clopidogrel/Telmisartan; § indicates Aspirin + Extended Release Dipyridamole/Placebo; | | indicates Clopidogrel/Placebo.

**Table 3:** Adjusted HRs (95% CIs) for all-cause mortality following an ischemic stroke in relation to categorical indicators of BMI and diabetes.

**All-Cause Mortality**	**Obesity Categories**					
	Underweight/normal weight		Overweight		Obese	
	Deaths/total	HR (95% CI)	Deaths/total	HR (95% CI)	Deaths/total	HR (95% CI)
**Diabetes**						
No	495/5,958	1.00	337/5,881	0.70 (0.61, 0.81)	126/2,693	0.54 (0.44, 0.66)
Yes	219/1,906	1.47 (1.24, 1.73)	180/2,257	0.95 (0.73, 1.13)	128/1,551	0.98 (0.80, 1.21)
Interaction (additive): RERI *∗* (95% CI),			-0.221 (-0.499, 0.057),		-0.019 (-0.319, 0.280)	
AP † (95% CI)			-0.234 (-0.327, -0.140)		-0.020 (-0.112, 0.073)	
Interaction on multiplicative scale: p-value			*P*=0.5094		*P*=0.1440	

HRs are adjusted for age, gender, race/ethnicity, qualifying stroke neurological severity, ischemic stroke subtype, baseline systolic blood pressure, hypertension, treatment assignment, history of congestive heart failure, history of atrial fibrillation, history of coronary artery disease, history of previous stroke or TIA, history of myocardial infarction, smoking status, alcohol consumption, and average physical activity prior to qualifying stroke.

*∗*RERI: relative excess risk due to interaction; †AP: attributable proportion due to interaction.

**Table 4:** Adjusted HRs (95% CIs) for cardiovascular-related mortality following an ischemic stroke in relation to categorical indicators of BMI and diabetes.

**Cardiovascular-Related Mortality**	**Obesity Categories**					
	Underweight/normal weight		Overweight		Obese	
	Deaths/total	HR (95% CI)	Deaths/total	HR (95% CI)	Deaths/total	HR (95% CI)
**Diabetes**						
No	288/5,598	1.00	190/5,881	0.72 (0.60, 0.88)	67/2,693	0.55 (0.41, 0.72)
Yes	136/1,906	1.53 (1.23, 1.89)	111/2,257	1.06 (0.85, 1.34)	95/1,551	1.44 (1.12, 1.86)
Interaction (additive): RERI*∗* (95% CI),			-0.187 (-0.561, 0.187),		0.372 (-0.064, 0.808)	
AP † (95% CI)			-0.176 (-0.296, -0.055)		0.258 (0.155, 0.361)	
Interaction on multiplicative scale: p-value			*P*=0.8118		*P*=0.0049	

HRs are adjusted for age, gender, race/ethnicity, qualifying stroke neurological severity, ischemic stroke sub-type, baseline systolic blood pressure, hypertension, treatment assignment, hyperlipidemia, history of coronary artery disease, history of previous stroke or TIA, history of myocardial infarction, smoking status, and average physical activity prior to qualifying stroke.

*∗*RERI: relative excess risk due to interaction; †AP: attributable proportion due to interaction.

## Data Availability

This work was facilitated by the Boehringer Ingelheim Policy of Transparency and Publication of Clinical Study Data, under which researchers may access and analyze clinical trial data with appropriate analytical tools. BIPI was given the opportunity to review the manuscript for medical and scientific accuracy as well as intellectual property considerations.

## Supplementary Materials

Supplementary 1 Supplementary Table 1: adjusted HRs (95% CIs) for all-cause mortality following an ischemic stroke in relation to categorical indicators of BMI and diabetes among current smokers.

Supplementary 2 Supplementary Table 2: adjusted HRs (95% CIs) for all-cause mortality following an ischemic stroke in relation to categorical indicators of BMI and diabetes among former/never smokers.

Supplementary 3 Supplementary Table 3: adjusted HRs (95% CIs) for cardiovascular-related mortality following an ischemic stroke in relation to categorical indicators of BMI and diabetes among current smokers.

Supplementary 4 Supplementary Table 4: adjusted HRs (95% CIs) for cardiovascular-related mortality following an ischemic stroke in relation to categorical indicators of BMI and diabetes among former/never smokers.
