# Supplementary material for: Determining the Joint Effect of Obesity and Diabetes on All-Cause Mortality and Cardiovascular-Related Mortality following an Ischemic Stroke
Source: Stroke Res Treat. 2018 Aug 9;2018:4812712. doi: 10.1155/2018/4812712 (PMC6106950; doi:10.1155/2018/4812712)
Supplement: Supplementary 3 — Supplementary Table 3: adjusted HRs (95% CIs) for cardiovascular-related mortality following an ischemic stroke in relation to categorical indicators of BMI and diabetes among current smokers. [file 4812712.f3.docx]

**Supplementary Table 3. Adjusted HRs (95% CIs) for Cardiovascular-Related Mortality following an Ischemic Stroke in Relation to Categorical Indicators of BMI and Diabetes among Current Smokers.**

| **Cardiovascular-Related Mortality** | **Obesity Categories** | | | | | |
| --- | --- | --- | --- | --- | --- | --- |
|  | Underweight/normal-weight | | Overweight | | Obese | |
|  | Deaths/ total | HR  (95% CI) | Deaths/ total | HR  (95% CI) | Deaths/ total | HR  (95% CI) |
| **Diabetes** |  |  |  |  |  |  |
| No | 75/1,682 | 1.00 | 35/1,217 | 0.70  (0.46, 1.06) | 9/406 | 0.57  (0.28, 1.16) |
| Yes | 26/362 | 1.72  (1.09, 2.73) | 18/427 | 1.11  (0.65, 1.88) | 15/194 | 2.26  (1.25, 4.07) |
| Interaction (additive): RERI* (95% CI),  AP † (95% CI) | | | -0.310 (-1.242, 0.622),  -0.281 (-0.581, 0.019) | | 0.965 (-0.472, 2.402)  0.427 (0.196, 0.659) | |
| Interaction on multiplicative scale: p-value | | | *P*=0.8328 | | *P*=0.0865 | |

HRs are adjusted for age, gender, race/ethnicity, qualifying stroke neurological severity, ischemic stroke sub-type, baseline systolic blood pressure, hypertension, treatment assignment, hyperlipidemia, history of coronary artery disease, history of previous stroke or TIA, history of myocardial infarction, and average physical activity prior to qualifying stroke.

*RERI = relative excess risk due to interaction, †AP=attributable proportion due to interaction
